# PHROG: A Multimodal Feature for Place Recognition

**DOI:** 10.3390/s17051167

**Published:** 2017-05-20

**Authors:** Fabien Bonardi, Samia Ainouz, Rémi Boutteau, Yohan Dupuis, Xavier Savatier, Pascal Vasseur

**Affiliations:** 1 Laboratoire d’Informatique, de Traitement de l’Information et des Systèmes, Normandie University, UNIROUEN, UNIHAVRE, INSA Rouen, LITIS, 76000 Rouen, France; samia.ainouz@insa-rouen.fr (S.A.); pascal.vasseur@univ-rouen.fr (P.V.); 2 Institut de Recherche en Systèmes Électroniques Embarqués, Normandie University, UNIROUEN, ESIGELEC, IRSEEM, 76000 Rouen, France; remi.boutteau@esigelec.fr (R.B.); xavier.savatier@esigelec.fr (X.S.); 3 Centre d’Études et d’Expertise sur les Risques, l’Environnement, la Mobilité et l’Aménagement, CEREMA, 76000 Rouen, France; yohan.dupuis@cerema.fr

**Keywords:** feature extraction, cross-spectral imaging, scene matching, visual place recognition

## Abstract

Long-term place recognition in outdoor environments remains a challenge due to high appearance changes in the environment. The problem becomes even more difficult when the matching between two scenes has to be made with information coming from different visual sources, particularly with different spectral ranges. For instance, an infrared camera is helpful for night vision in combination with a visible camera. In this paper, we emphasize our work on testing usual feature point extractors under both constraints: repeatability across spectral ranges and long-term appearance. We develop a new feature extraction method dedicated to improve the repeatability across spectral ranges. We conduct an evaluation of feature robustness on long-term datasets coming from different imaging sources (optics, sensors size and spectral ranges) with a *Bag-of-Words* approach. The tests we perform demonstrate that our method brings a significant improvement on the image retrieval issue in a visual place recognition context, particularly when there is a need to associate images from various spectral ranges such as infrared and visible: we have evaluated our approach using visible, Near InfraRed (*NIR*), Short Wavelength InfraRed (*SWIR*) and Long Wavelength InfraRed (*LWIR*).

## 1. Introduction

Nowadays, cameras are widely used in outdoor robotics for tasks such as localization and mapping. To ensure an efficient navigation in its environment, a robot needs a well-estimated localization at all times, and ideally its navigation method should fit different sensors and not rely on an exclusive one. Besides, we can imagine a fleet of agents evolving in the same area, with various sensor devices: in a collaborative way, each agent should be able to share its map and to use map information coming from others agents [1]. For such a purpose, chosen image descriptors have to be suitable to each sensor and repeatable with others sensors.

A part of the localization problem based on computer vision refers to place recognition: some images from a memory obtained during a first experience, called key-frames, are matched with images acquired on the fly. This process serves to estimate at first an approximated position such as described in [2,3]. This step aims to reduce the drift produced in SLAM (*Simultaneous Localization And Mapping*) or to increase localization accuracy [4]. The visual place recognition process is also used as the *loop closure* process as presented by Chapoulie in [5]. However, due to perceptual-aliasing in outdoor environments as well as illumination, weather and seasonal variations, several contributions show that this matching process is especially tricky [6,7,8].

In this paper, we offer an overview of the recent solutions put forward to handle the image retrieval belonging to a visual place recognition process with a set of multimodal sensors. We propose a new feature descriptor which improves results presented in the literature. We complement this new descriptor definition with experimental results on local features applied to the long-term localization problem with a Bag-of-Words approach and establish a benchmark including our algorithm and the most popular ones. We show that our proposal outperforms the usual methods on datasets with important constraints generated by images acquired in distinct spectral ranges (visible-NIR, visible-SWIR and visible-LWIR).

We itemize our contributions as follows: firstly (Section 2), we lead an analysis of the different state-of-art approaches facing multimodal datasets in the context of visual place recognition. In a second phase (Section 3), we give details on the feature extractor we design and the reasons which have led to such choices. In a third part (Section 4), we confront our proposal to several datasets used in the visual place recognition community and an additional one we acquired to push further the constraints to the limits. In a final part (Section 5), we draw conclusions on the works done and their needs for future improvements.

## 2. Related Work

Classical approaches for localization, based only on visual data, deal with the same outline as image retrieval frameworks: firstly, one or several chosen features are extracted from images, and then an algorithm identifies the most discriminant data (mostly invariant to changes such as illumination), and lastly the discriminant data is compared on the basis of a chosen metric (for example L1 distance). Features used in the visual localization framework can be divided into two main categories: global image descriptors and local features [2].

### 2.1. Images and Intermediates Representations

Several emerging methods rely directly on raw data from the camera. As a matter of fact, visual sensors are particularly sensitive to high dynamic appearance changes in outdoor environments (changes inherited from the illumination of the scene like sun visibility, shadows, etc.). A relevant strategy performs a preprocessing step on raw images called illumination invariant transform before computing a global description [9]. Some other authors choose as well global description of the images, called image signatures, and compare directly these compact representations [6,8]. These methods give interesting results but require that the query image and stored images share a very close point of view.

Local features, or point features, are heavily used in computer vision applications. Some benchmarking on local features for general applications has already been done: reference [10] for instance or more recently [11] which emphasizes evaluation on fast *binary features*. Compared to global image descriptors, features points have some advantages. They can be used later for an estimation of relative pose between two cameras through the computation of the fundamental or essential matrices as explained in [12], and even to estimate a sparse 3D structure of the environment thanks to triangulation (further explanations in [13]). These methods remain the core process of various SLAM systems as depicted in a survey made in [14]. Nevertheless, the size of the data extracted from images depends on the number of detected feature points whereas global image signatures have a fixed size.

A recent survey classifies Bag Of Words techniques as a third category [15]. Bag Of visual Words (*BoW*) is one of the most common approach in robotics. The BoW approach is the result of an analogy with words in textual retrieval: concepts developed for large full-text search engines are extended to image retrieval in large database and object recognition [16,17]. Each image is represented as a document containing words (the features). The set of visual words, or statistics obtained from this set, is therefore coded as an intermediates representation of the image information, which is sometimes called *Mid-Level features* [18]. A request on a corpus can be executed with one or several words from the set and a ranking is established according to their distinctiveness across the documents. Bag Of Words and these techniques are globally efficient, but their complexity can be a burden for embedded systems and fast computation requirements.

For a few years, the evolution of hardware capabilities, particularly GPU technologies, has incited the revival of neural networks recipes. The computer vision community is showing a growing interest to learned features thanks to improvements made on deep neural networks and their convolutional variants. The dawn of deep learning on images tends to use full images as inputs and let the system learn by itself an empirical model. The results-oriented deep learning approaches have brought relevant performances on several common computer vision and classification problems. Results presented in [19,20,21] for example outperform the previous methods. Some other authors substitute a smaller part of the computer vision process: for example in [22], the author replaced only the feature description and the metric steps by a deep neural network, compositing hence an implicit intermediates representation.

Nonetheless, deep learning attractiveness has major counterparts and suffers from non-negligible drawbacks: deep convolutional neural networks are extremely greedy and require both huge datasets to make the learning process succeed and massive computational resources. These holistic approaches indeed are still seen as “black-boxes” and the underlying theory is an active research domain.

### 2.2. Features and Multimodality

Another source of problems may rise due to the diversity of sensors which leads to various images of the real scene. As a matter of fact, different visual sensors have been used for the V-SLAM (*Visual-SLAM*) task in the literature. For example, monoSLAM described in [23] proposes the use of a single camera. In [24], the authors have developed a similar method with infrared monocular camera. This sensor change raises another issue known by the community as *multimodality*. In addition to the need of an invariance to appearance changes discussed above, the matching process has to be extended further between data coming from sensors having different spectral ranges. Multimodal image registration is a well documented research area for medical and aerial imaging but is quiet recent in robotics domain.

Dedicated to visual servoing, the method described in [25] is a good example which focuses on mutual information on whole images for registration and tracking. In [26], the authors use multimodal image registration at the heart of a SLAM process. Reference [27] also proposes a multimodal approach involving visible and thermal imaging systems and claims its performances in daylight as well as at night. Nevertheless, both make use of two spectral ranges at the same time, that means using the same mix of information coming from a particular set of sensors at all times. In this paper, we put forth assumptions that the sensor set can change over time or between different agents and thus, the image retrieval process should be tolerant to these changes.

Some other methods also deal with multimodality but instead of adapting algorithms from medical imaging, they rather start from standard descriptors as SIFT or SURF usually used in computer vision: Ricaurte et al. for instance evaluate feature points efficiency on both visible and infrared spectra [28]. Other authors make further modifications on usual descriptors and claim that these new descriptors are more robust to multimodal matching [29,30].

### 2.3. Visual Place Recognition Supervision

The image retrieval process is usually wrapped in an overall decision algorithm in the context of a SLAM system: if the vision subsystem suggests some faulty image associations, these could be balanced by an estimated position in a map and refined over the next sensors’ acquisitions. The survey [2] gives details on maps and decision processes used in SLAM. The works presented here keep the focus only on the image retrieval process without an overall reconsideration.

The most recent and probably closest publication to our work is [22]: in this paper, the authors make the decision of substituting the feature points description and metric evaluation steps by different Convolutional Neural Networks (*CNN*). They show that one of the proposed *CNN* provides slightly better results than the traditional methods facing multimodality. However, they handle the feature point detection with various algorithms, *SIFT* (*Scale Invariant Feature Transform*) [31] and *FAST* (*Features from Accelerated Segment Test*) [32], and various hand tuned parameters and state that it is still an open issue. In this way, their *CNN* has been trained and evaluated on datasets whose image pairs are rectified (same pixels resolution and strictly the same point of view). In our work, we make the hypotheses that point of view of the same scene could vary. Furthermore, we evaluate our approach on datasets with major appearance changes in order to estimate its robustness against long-term changes. In this paper, we justify the use of Harris feature detector and propose a new feature descriptor which improves experimental results dedicated to the visual place recognition problem and its extension to multimodality. Compared to most papers which lead tests on two modalities (and sometimes three), we propose a comparison between visible, near infrared (*NIR*), short-wavelength infrared (*SWIR*) and long-wavelength infrared (*LWIR*).

## 3. Methodology

**Figure 1:**
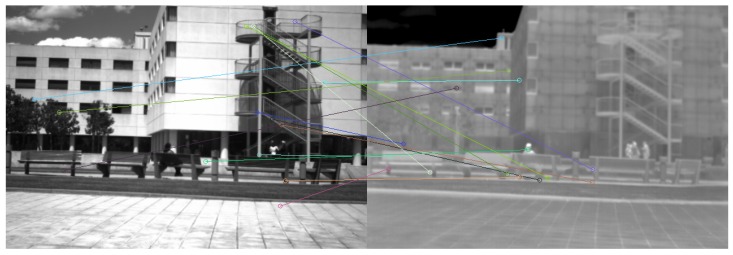
Fundamental matrix calculation made with extracted SIFT features. The numerous faulty matchings pairs do not allow the algorithm to converge to a consistent result.

A traditional method in robotics to estimate a camera pose is the computation of the fundamental matrix [13]. Thanks to a RANSAC (*RANdom SAmple Consensus*) strategy, this computation allows to eliminate faulty feature point matchings. In Figure 1, we give an example of the remaining matched SIFT points after the computation of the fundamental matrix. The SIFT descriptors disparity between two spectral ranges is such that the algorithm does not converge to a satisfying solution. For that reason, we opt for a procedure which is more widespread in the image retrieval community.

**Figure 2:**
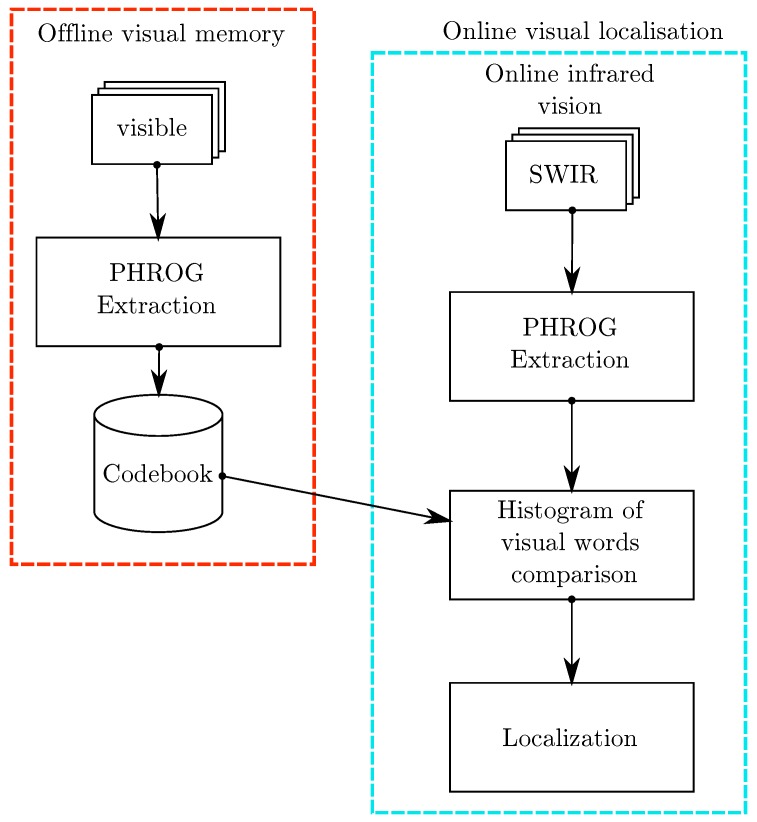
Overview of the method: PHROG features are extracted from a whole sequence considered as the memory and a codebook is computed. Each image from a live sequence is then compared consecutively with other images according to their histogram of visual words.

We detail our work in the following parts: in Section 3.1, we highlight the feature extraction process and its sub-steps. We focus on the study of two main approaches to detect points of interest in images in Section 3.2. We show that corner detectors are more suitable to multimodal datasets than patch-based methods such as Differences-of-Gaussians. We draw the relevant consequences and propose a new approach to describe the corners’ neighborhood, we call PHROG (Plural Histograms of Restricted Oriented Gradients). We introduce the multiscale approach in Section 3.3. Specificities are given on the description pattern used in Section 3.3 and how we extract information with a HOG-like description in Section 3.4. In Section 3.5, we explain how a few additional computational steps can improve the features similarity evaluation. Finally, Section 3.6 presents explanation on the Bag-of-Words retrieval process we use. The method overview is represented in Figure 2.

### 3.1. Features Extraction: Detection and Description

A usual way to extract relevant information from images is to choose a particular feature type: features can be points, regions, edges or straight lines for example. Point-like interest operators are the most common ones in SLAM or “Structure-From-Motion” applications mainly because they allow further computation of geometrical relations between several images or a sparse 3D reconstruction of the surrounding environment. Many different feature extractors methods have been proposed in the literature. The computational process is usually divided into two parts: detection and description of the feature point. The reader may refer to two surveys on detectors [33,34], a study on local descriptors [10] and a comparative study on binary descriptors [11] to get practical details on the different algorithms involved. Binary descriptors have been preferred for their computational speed, for a few years, but face the “gradient reversal” problem raised by multimodality as explained in Section 3.4.

### 3.2. Preliminary Tests on Feature Point Detectors Repeatability

Historically, the first feature points detector methods were corner detectors: they use the close neighborhood of each pixel to distinguish between corners and irrelevant pixels. References [35,36] compute the “cornerness” map of an image thanks to the computation of auto-correlation matrices and criterion on their eigenvalues. Later, studies have been led on detectors with the ability to estimate a feature and its appropriated “scale” for description in order to enhance feature recognition even if the viewpoint has changed. It results in methods like SIFT [31] or SURF [37] that respectively use Difference-of-Gaussians or an approximated formula to find a feature and its scale. The features are then closer to “patches of interest” rather than corners. These sets of connected pixels are generally called *blobs*.

**Figure 3:**
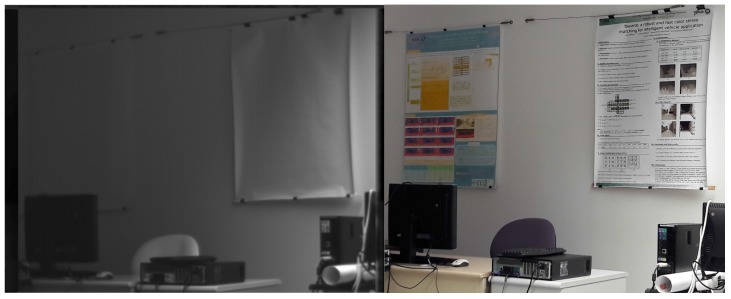
Serigraphy in visible and infrared spectra: whereas the printed shapes are discernible in the visible spectrum, the posters seem blank in the infrared spectrum.

**Figure 4:**
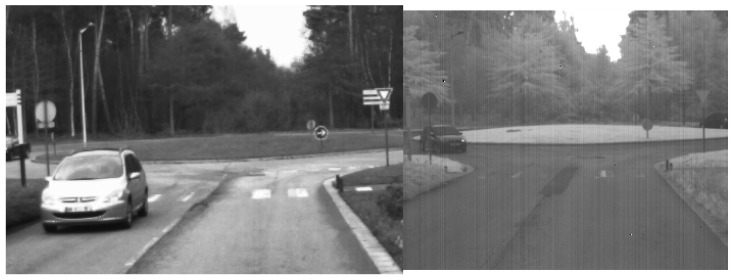
A infrared-visible pair from our dataset introduced in Section 4.5.

As observed in [22], objects in infrared images look different than in color images. Objects globally loose their textures: for example, a screen printing with many colors and shapes seems homogeneous in infrared images (Figure 3). Another noteworthy example is that of vegetation which appears much darker in visible imagery than in infrared one (a landscape looks “snowy” as in Figure 4).

**Figure 5:**
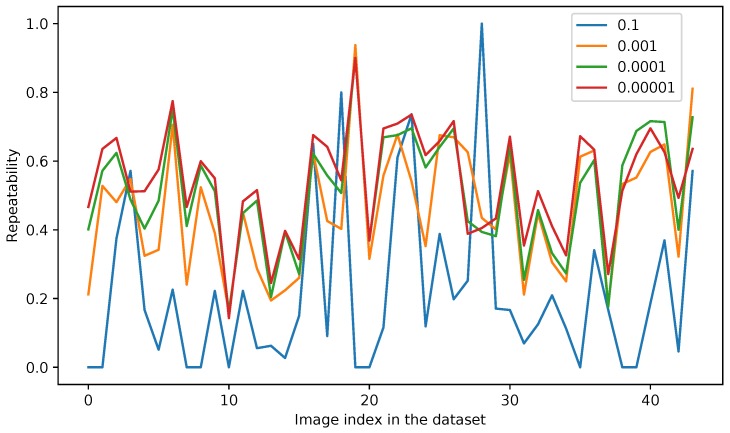
Detector parameters tuning: repeatability for several “quality levels” according to each image pair in the dataset.

**Figure 6:**
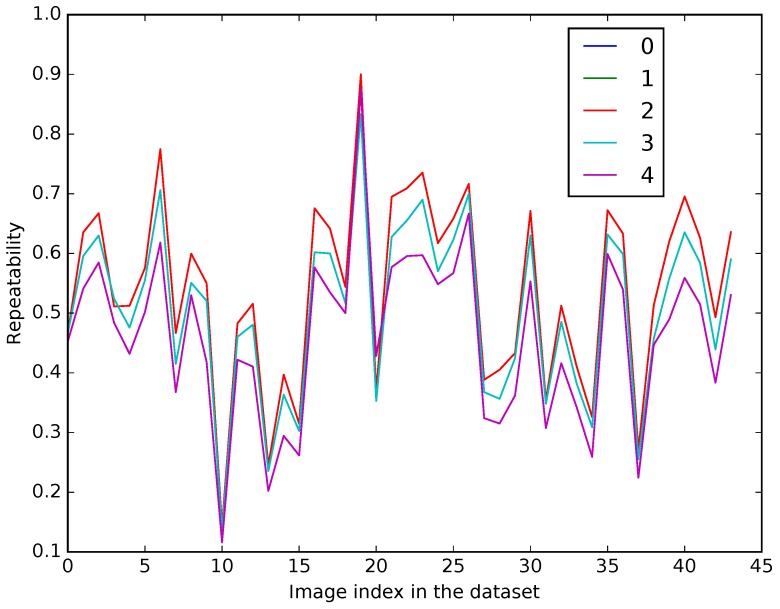
Detector parameters tuning: repeatability for different minimal distances (in pixels) ccording to each image pair in the dataset.

Knowing what happens globally to textures across different spectral ranges, we have decided to validate the detection step itself in order to choose the best detection approach, namely corner detection or patch of interest detection. We have taken pairs of images from the visible-LWIR dataset introduced in [38]. Each pair has been adjusted in order to make viewpoints and resolutions of both images (infrared and visible) exactly the same. We thus ran detection algorithms for each modality and checked if the features are found in both modalities. We focused our tests on Harris corner detector and Difference-of-Gaussians as implemented in the SIFT OpenCV library (http://opencv.org/releases.html). We have considered the repeatability as the evaluation criterion, which is given, for each pair, as a ratio between the number of points of interest detected at the same position in both images, to the total number of points of interest returned by the algorithm in both images. The parameters of each algorithm have been tuned to obtain the best results according to the following method: we have computed the repeatability on a full sequence with several values for each detector parameter. Figure 5 and Figure 6 show the obtained results with Harris detector when tuning respectively the *quality level* and the *minimal distance* between two contiguous detected points. The tuning of the quality ratio can provide significant improvements on repeatability for some image pairs. Globally results are better when a low quality level (equal to 0.0001) is chosen. Minimum distance between two pixels selected as features is a less determinant criterion on repeatability and we have chosen to keep a minimal distance of 2 pixels in order to detect less points while preserving the repeatability.

**Figure 7:**
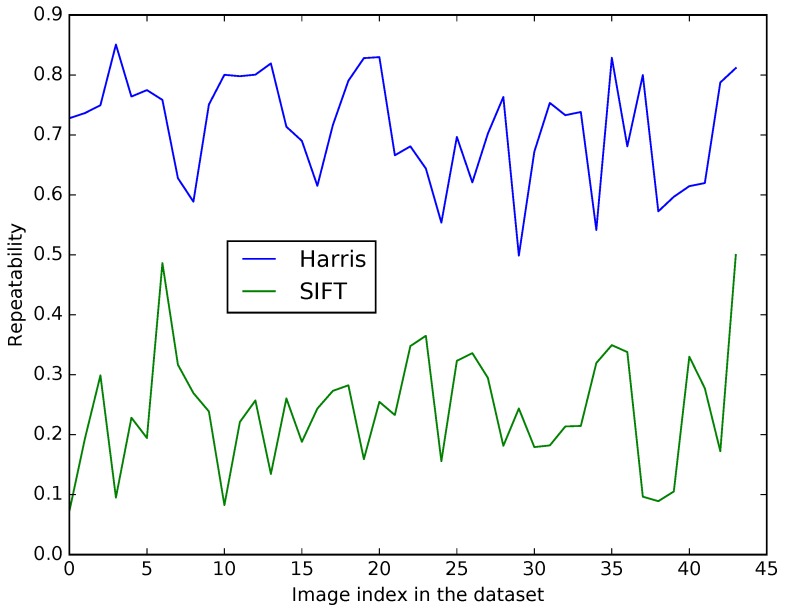
Detectors repeatability on LWIR dataset according to each image pair in the dataset.

We define a tolerance of two pixels in positions returned by each algorithm. Figure 7 gives the repeatability results according to each pair of the visible-LWIR dataset [22]. For each image pair, the repeatability is far better with a corner-like detection approach. We assume that these results are due to the fact that object’s shapes are mostly the same in both modalities whereas textures tend to differ. Corner-like features conception is more correlated to the object’s shapes than patch-like ones. Hence we have chosen to use the Harris method as the detector procedure.

### 3.3. Multiscale Description Pattern

**Figure 8:**
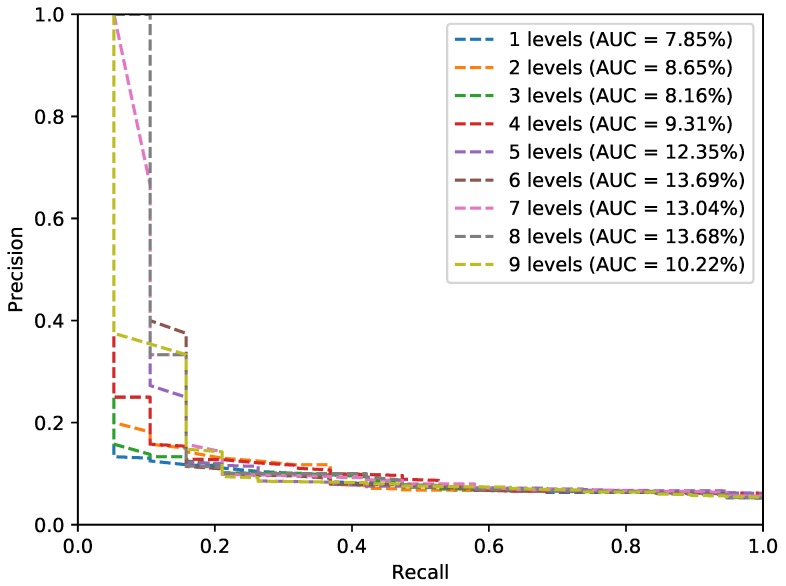
Precision-Recall curves and related Area Under The Curves (*AUC*) according to the number of levels description used in PHROG when applied on the VPrice dataset.

Given the Harris nature, we have no prior information concerning the scale of the image patch to describe. Hence, we have conceived to establish several descriptions at different scale levels. We call this approach PHROG. As done by scale invariant features like *SIFT*, we compute a Pyramid of Gaussian for that purpose: the first scale level is the original image. To compose next levels, we convolute the source image with a 5×5 Gaussian kernel to smooth and remove its high-frequency components. We then down-sample the resulting image by taking one pixel out of every two in both directions. We make new iterations like this according to the desired scale level number. During our experimentations, as we get images from sensors having different resolutions and with varying viewpoints of the scene, we evaluate the efficiency of PHROG with several numbers of scale description. We use Precision-Recall curves and their AUC (*Area Under the Curve*) as an evaluation of the performance of PHROG with different scale parameters. We give, in Figure 8, an example of the results obtained on the VPrice dataset (which is introduced in Section 4.4). We find that 5 scale levels are appropriate because the computation of supplementary levels brings no significant benefits on matching results and is a good compromise for the memory cost of our proposal. We make no further corner refinement or additional image interpolation.

### 3.4. Gradient Direction Invariant HoG Features

**Figure 9:**
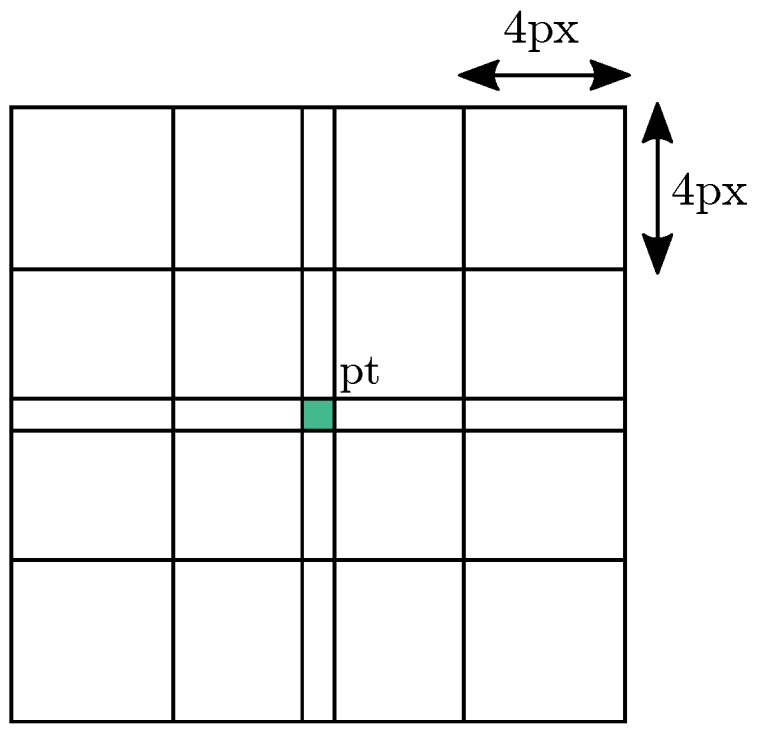
Pattern used during the description process. It defines areas to be extracted around a feature point.

On each level, we compose a descriptor inspired by SIFT pattern (cf. Figure 9): we consider a neighborhood of 4×4 areas which are 4×4 pixels sized. Central areas overlap each other so that corner pixel (the center position of the feature) is included in 4 areas and each pixel remaining at the same abscissa or ordinate belongs to 2 areas. Unlike SIFT descriptor, we make no additional weighting on the pixels intensity value before the following processing: given the overlapping area, the information from central pixels is already considered twice compared to the other pixels (even four times for the central pixel).

For each given area, we compute a *N*-bins Histogram of Oriented Gradients. The “gradient reversals” problem due to multispectral imaging is explained in [29]: a particular material has different reflectance properties according to spectral ranges such that two different materials may have varying responses when a scene is observed with several sensors. Particularly, an area of high contrast in an image due to the edges of an object of one material in front of another object with a different material may appear as its own negative with another sensor: white pixels in the first image appear as black in the second one and *vice versa*. If we compute HOG on both images, gradients will have the same orientation and approximately the same norm but an opposed direction.

**Figure 10:**
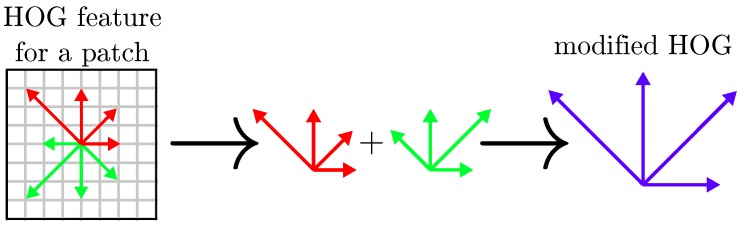
HOG feature to gradient reversals invariant HOG feature conversion: gradients with opposing direction are added. Descriptors are hence twice smaller.

In order to handle the gradient reversals, we restricted a traditional HOG descriptor to a half-size one of which opposed gradient directions are summed (Equations (1) and (2)). This concept is exemplified in Figure 10: in this way, we keep in the descriptor the gradient orientation information without its direction.

(1) $$h_{i}=\sum_{k}\alpha_{\theta_{k}}r_{k}$$

(2) $$\alpha_{\theta_{k}}=\left\{\begin{array}{cc} 1 & \mathrm{if}\theta_{k}\in \left[\frac{i}{N}\pi ,\frac{i+1}{N}\pi \right]\cup \left[\frac{i}{N}\pi +\pi ,\frac{i+1}{N}\pi +\pi \right]\\ 0 & \mathrm{else} \end{array}\right.$$

with *N*, the chosen number of bins in the histogram, $h_{i}$ the $i^{th}$ bin, $\theta_{k}$ the orientation of the gradient at pixel *k* and $r_{k}$ the magnitude of the gradient at pixel *k*. Coefficient $\alpha_{\theta_{k}}$ is equal to 1 when the orientation of the gradient at pixel *k* is included in the interval $\left[\frac{i}{N}\pi ,\frac{i+1}{N}\pi \right]$ or in its opposite direction (in $\left[\frac{i}{N}\pi +\pi ,\frac{i+1}{N}\pi +\pi \right]$).

### 3.5. Hellinger Kernel on Descriptors

It has been proved in several studies that using Euclidean distance is not the best practice to compare features which carry information in the form of histograms. For these particular cases, $\chi^{2}$ or Hellinger measures are a better choice. In this way, authors from [39] propose some modifications on SIFT descriptor and called it RootSIFT. The idea behind this tweaking is that comparing RootSIFT descriptors with the help of Euclidean distance is the same than applying the Hellinger kernel on original SIFT descriptors. We apply the same process on our histograms of gradients: given $x_{1}$ and $x_{2}$ two vectors with unit Euclidean norm (${∥x_{i}∥}_{2}=1$), their Euclidean distance is given by Equations (3) and (4):

(3) $$d_{Eucl}\left(x_{1},x_{2}\right)={∥x_{1}-x_{2}∥}_{2}=\sqrt{{∥x_{1}∥}_{2}^{2}+{∥x_{2}∥}_{2}^{2}-2{x_{1}}^{T}x_{2}}$$

(4) $$d_{Eucl}\left(x_{1},x_{2}\right)=\sqrt{2-2K_{Eucl}\left(x_{1},x_{2}\right)}$$

where $K_{Eucl}\left(x_{1},x_{2}\right)={x_{1}}^{T}x_{2}$ is the Euclidean kernel (or similarity). We would like to change this similarity by the Hellinger kernel given in Equation (5):

(5) $$K_{Hell}\left(x_{1},x_{2}\right)=\sum_{j=1}^{N}\sqrt{{x_{1}}_{j}{x_{2}}_{j}}$$

for $x_{1}$ and $x_{2}$ two L1 normalized histograms ($\sum_{j=1}^{N}{x_{i}}_{j}=1$ and ${x_{i}}_{j}\geq 0$). An easy way to compute Hellinger similarity on descriptors is to normalize the histograms vectors and square root each histograms element. Thus, $K_{Eucl}\left(\sqrt{x_{1}},\sqrt{x_{2}}\right)={\sqrt{x_{1}}}^{T}\sqrt{x_{2}}=K_{Hell}\left(x_{1},x_{2}\right)$ and using the Euclidean distance on these preprocessed descriptors is equivalent to making use of the Hellinger similarity on initial descriptors.

### 3.6. Bag-of-Words Retrieval

**Figure 11:**
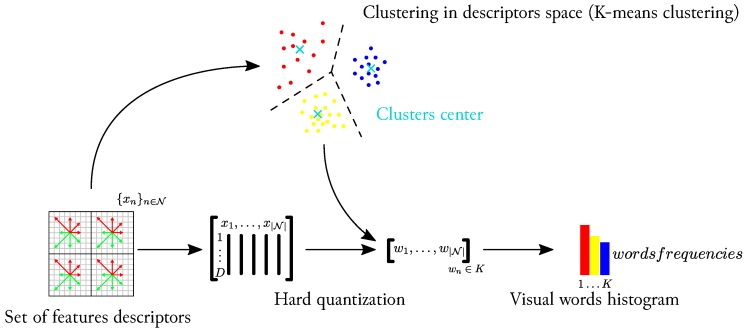
Bag-of-Words approach: a “learning dataset” is first used to cluster extracted features in the descriptor space. Features are then quantified according to nearest cluster. The resulting representation of the images are visual words histograms.

A common strategy used for image retrieval is to compute a Bag-of-Words as explained in [16]. Used data are divided into two sets: the first composes the memory and the second one is referred to as “live” or “online” sequence because this information is usually acquired progressively during the localization process. Bag-of-Words retrieval takes advantage of a preprocessing step applied on images from the memory part of the dataset. A global schema of the Bag-of-Words process is presented in Figure 11. All feature points are first extracted from all images from the memory. A *K-means* algorithm then separates the whole descriptors space in *K* clusters (from 1000 to 8000 clusters according to the different test cases). The set of each cluster’s mean descriptor is outlined as the *vocabulary*. All the descriptors are then quantized relatively to this vocabulary. Each *word* of the vocabulary is subsequently weighted by a *TF-IDF* score (*Term Frequency-Inverse Document Frequency*). The calculation of TF-IDF scores is a product of two terms:

- ${\mathrm{tf}}_{i,j}$ (*Term Frequency*) is defined as follows (Equation (6)):
  (6) $${\mathrm{tf}}_{i,j}≜\frac{n_{i,j}}{\sum_{k=1}^{\left|I\right|}n_{k,j}}$$
  where $n_{i,j}$ is the number of occurrences of the word having index *i* in the dictionary, in the image having index *j* in the memory database, and |I| is the total number of images in the corpus.
- ${\mathrm{idf}}_{i}$ (*Inverse Document Frequency*) is defined as follows (Equation (7)):
  (7) $${\mathrm{idf}}_{i}≜\log\frac{\left|I\right|}{\left|\{i_{j}:w_{i}\in i_{j}\}\right|}$$
  where |I| is the total number of images in the corpus and $\left|\{i_{j}:w_{i}\in i_{j}\}\right|$ is the number of images where the word $w_{i}$ occurs.

Thus, scores ${\mathrm{tf}-\mathrm{idf}}_{i,j}={\mathrm{tf}}_{i,j}\times {\mathrm{idf}}_{i}$ allow us to specify words which are the most relevant both in a given image from the memory and in the whole database. During the processing of the “on-line” sequence, we extract features from each frame and quantify them relatively to the dictionary computed with memory sequence. The most relevant words found in the current image figure out the closest image from the memory.

## 4. PHROG Applied to the Visual Place Recognition Problem

As mentioned in the introduction, several works and developments have been made to deal with the long-term visual place recognition problem. These works focus on two main approaches: the first one concerns with the image description itself and tried studies to improve the image matching one by one. The other way considers a sequence and not only a single image. Thus, temporal coherence allows to envision a filtering on faulty image matching. Works presented in this paper remain to the first one. Our aim is to improve the matching of two images of the same scene when sensors with different spectral ranges are used. In order to evaluate our method and usual well-known methods in the literature, we use several datasets. Some come from recent available anterior works in the community. We have prepared our own additional dataset to evaluate the different techniques against increased constraints and difficulties.

**Figure 12:**
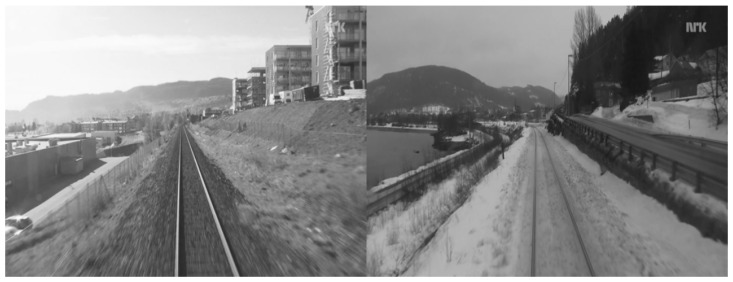
An example when the algorithm fails. The aliasing between the queried image (left side) and the image in memory returned by the algorithm is major.

For each test case, we take one modality from a dataset (being visible or infrared) and build a codebook off-line. We refer to this subset as the “memory”. After a few experimentations, we have determined that codebooks composed of 1000 words are a good compromise between efficiency and computing speed. We refer to the other subset as the “live” sequence. It is composed of images from the other modality composing the dataset. In other words, for each test case, the codebook is built thanks to a single modality and the image retrieval step is always performed with another modality. We try to match each image composing the live subset by founding the closest image in the memory according to the histograms of words in each image. If both memory image returned by the algorithm and live image come from the same pair, we consider the test as a true positive, else as a false positive. We experiment this method on the following test cases presented in the next parts. We give for each one the ratio between true positives and total number of live images in the dataset. We also show in Figure 12 an example where our proposed algorithm fails because of dominant aliasing between both scenes (shape of the railroad, building on the right side, background with mountains, etc.).

### 4.1. Experimental Setup

We have led the experimentations on a desktop computer running *Ubuntu 16.04 LTS* with an *Intel Core i7* processor and 8 GiBytes of RAM. We have limited to 10,000 the number of feature points detected in each frame. We have given details about the description pattern above (Figure 9), and the HOG modification. A PHROG descriptor vector is thus 2 times smaller (64 values) than a SIFT one. With such parameters, computing a codebook of 1000 visual words lasts between one hour and two hours depending on the considered dataset.

### 4.2. Experimentations on Visible and Near-InfraRed Images

**Figure 13:**
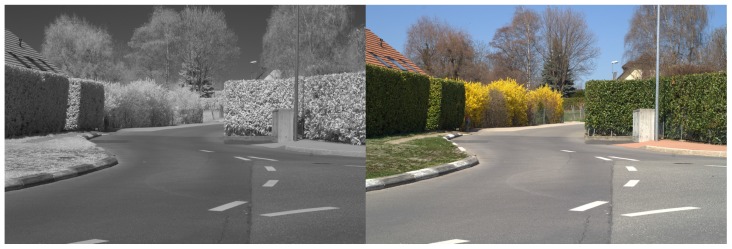
A Visible-NIR pair from the “EPFL” dataset.

The first dataset comes from EPFL (*École Polytechnique Fédérale de Lausanne* in Switzerland) and is presented in [40]. It is composed of several sorted subsets: *country*, *field*, *forest*, *indoor*, *mountain*, *oldbuilding*, *street*, *urban* and *water*. Each subset is composed of about 50 images pairs. Each pair is comprised of a visible image and a near-infrared (NIR) counterpart. Images from each pair have been rectified by the authors so that viewpoints and resolutions are strictly the same. A pair example is given in Figure 13. We choose to focus on *urban*, *street* and *country* subsets which are closer to robotics and navigation use-cases. The *country* subset was indeed used in [22] as the learning dataset for the whole experimentations. We choose alternatively the NIR images and the visible set as the memory and its live counterpart.

**Figure 14:**
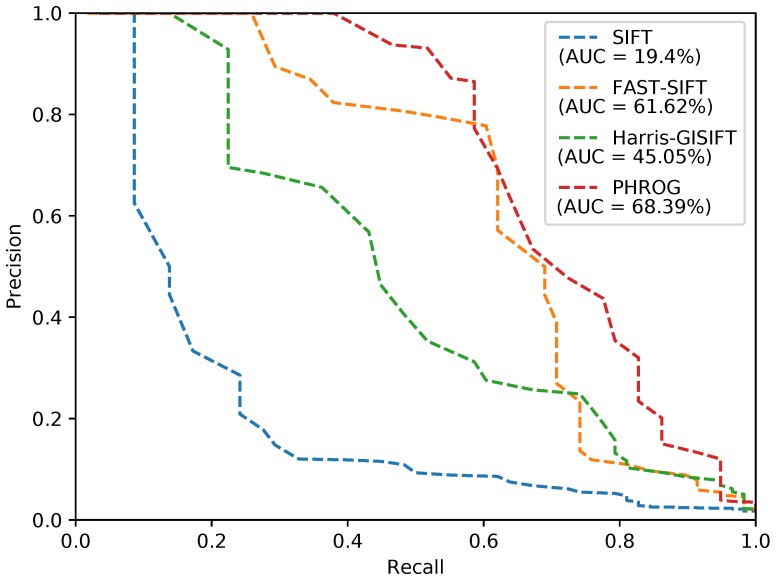
Precision-Recall curves from the “urban” dataset and AUC (*Area Under the Curve*) for each method.

**Figure 15:**
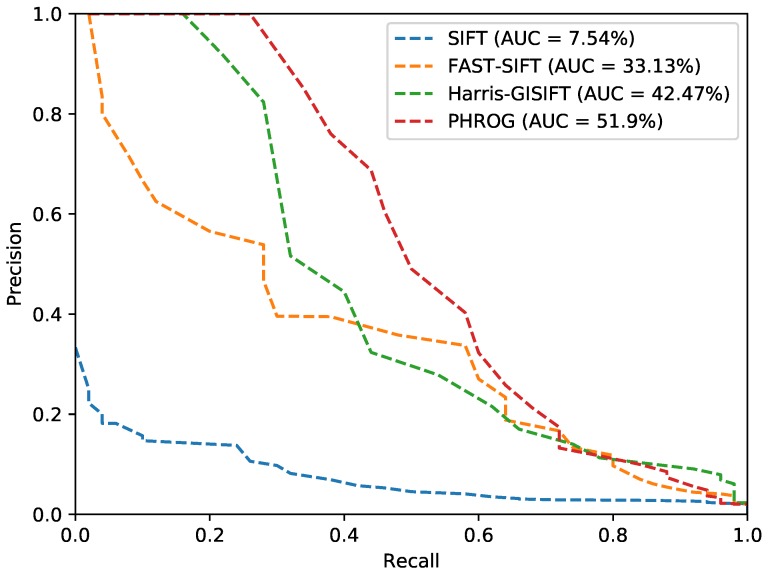
Precision-Recall curves from the “street” dataset and AUC (*Area Under the Curve*) for each method.

**Figure 16:**
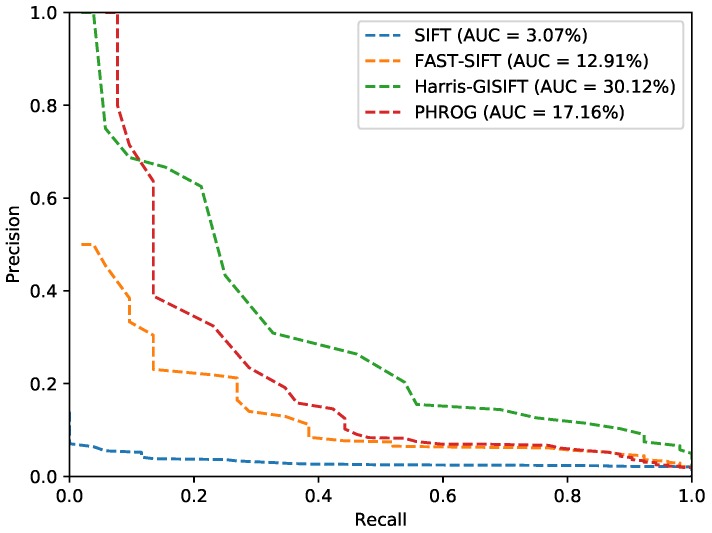
Precision-Recall curves from the “country” dataset and AUC (*Area Under the Curve*) for each method.

**Figure 17:**
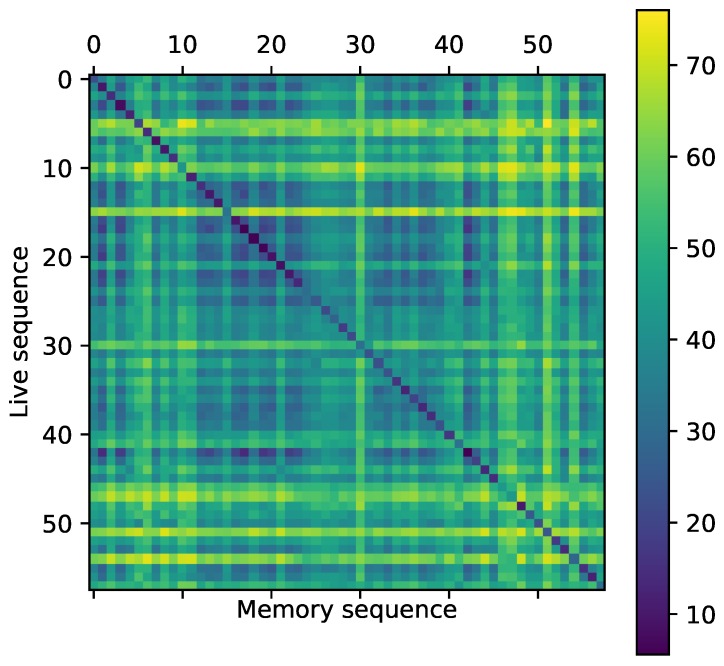
Confusion matrix between the memory sequence and the live sequence from the urban dataset obtained with PHROG. Values in the matrix are the distances computed for each possible image pair.

We give results in Table 1, Figure 14, Figure 15 and Figure 16 for each subset and detector-descriptor association and we show an example of a confusion matrix obtained on the *urban* dataset with PHROG (Figure 17). We can easily see that the diagonal of the confusion matrix presents the lowest distances between images: datasets have been synchronized in order to compute performance in a simple way. It allows us to consider that images associated on the diagonal are the truth and the others are distances computed for false matchings. This configuration of the confusion matrices makes possible the computation of Precision-Recall (*PR*) curves and their AUC (*Area Under The Curve*). PR curves have been widely used to evaluate search processes: a perfect classifier should present a PR curve going from (0,1) to (1,0) points with an area as close as possible to 1. With such consideration, the best method from a set is the one which presents the highest AUC.

As it can be seen, NIR images are not so different from visible images. It appears that image retrieval with traditional features gives very good matching ratios (see *urban* and *street* datasets). By the way, results on *country* dataset are more disparate: one possible explanation is that vegetation is much more present in this dataset and hence the gradient reversal problem appears more frequently than with building and inorganic matters. PR curves show that PHROG is the best on “urban” and “street” datasets: its AUC is higher by at least 9% than other evaluated methods. On the “country” dataset, PHROG does not hit the best AUC but its precision remains the best when recall is low: it is significant because it means that if the searching process returns only one result, PHROG gives the best answer.

### 4.3. Experimentations on Visible and Long-Wavelength InfraRed Images

**Figure 18:**
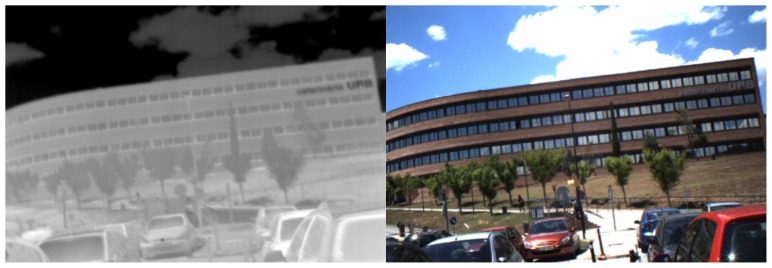
A Visible-NIR pair from the “Barcelona dataset”.

This part handles infrared images from a spectral range much further from visible spectrum than the previous one. We used the visible and Long-Wavelength InfraRed dataset introduced in [22,38]. This spectral range is appreciated for its thermal response and its possible usage as night vision system. This dataset encompasses outdoor view scenes of the Barcelona campus. Images from this dataset have also been rectified by their authors so that resolutions and viewpoints are the same. An instance from the dataset is given in Figure 18.

**Figure 19:**
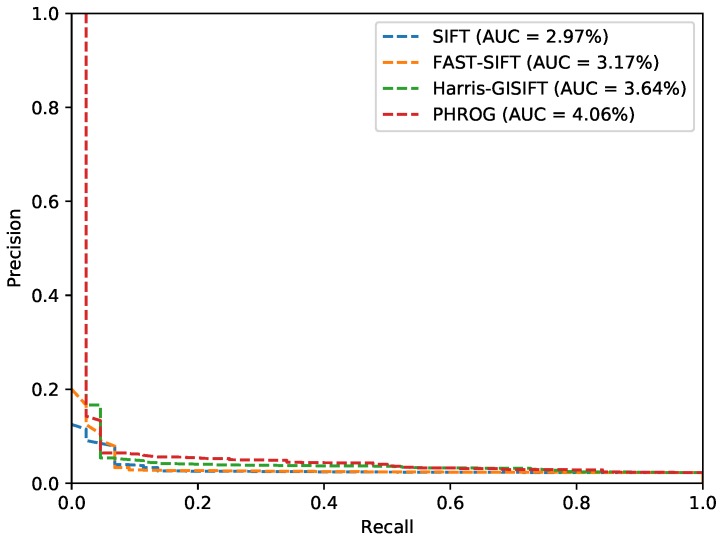
Precision-Recall curves from the “Barcelona” dataset and AUC (*Area Under the Curve*) for each method.

We try two situations by switching the modality used as the memory. The results are summed up in Table 2 and Figure 19 presents the PR curves. We can observe that matching ratio are slightly better when the LWIR subset is used as the source of the codebook composition. We suppose that less textured images enhance the computation of the visual words dictionary. Besides, our descriptor PHROG demonstrates its value on this dataset. PHROG gives significantly better results than the other methods with exactly the same parameters than on the EPFL dataset. However, we can note that AUC are very low (below 5%), even if the precision of PHROG is good when the recall is low. It means that the distances between images for true and false matchings are very close from each other but discriminant enough when there is a need to retrieve only one image. The further spectral ranges are, higher the differences between two frames are and the less efficient the usual methods become.

### 4.4. Experimentations on VPrice Dataset

**Figure 20:**
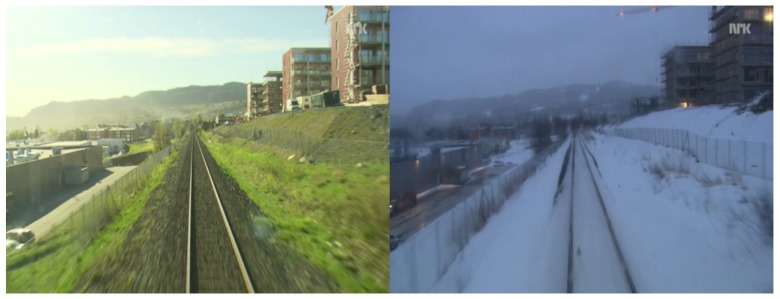
An image pair from the “VPrice dataset”.

**Figure 21:**
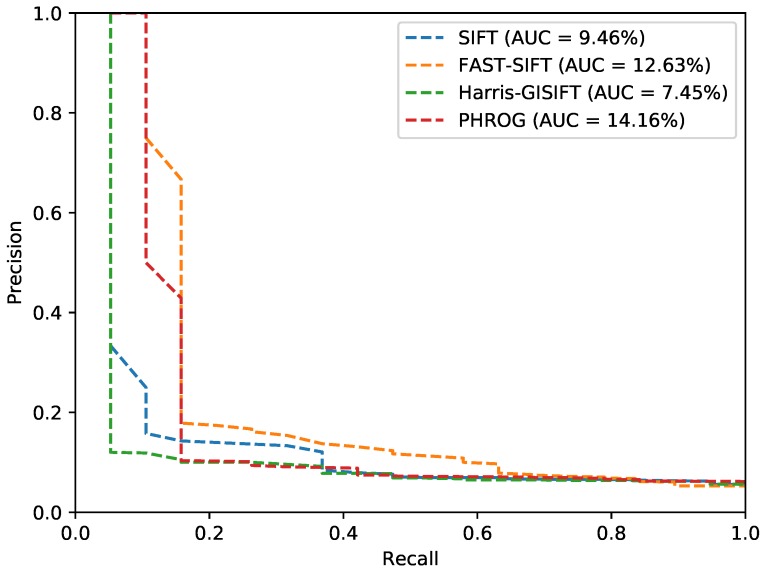
Precision-Recall curves from the “VPRiCE” dataset and AUC (*Area Under the Curve*) for each method.

In this part, we use a dataset which is composed of images picked up from the VPrice dataset (https://roboticvision.atlassian.net/wiki/pages/viewpage.action?pageId=14188617); an example from this dataset is given in the Figure 20. It has been designed to evaluate long-term place recognition. Both sequences from the dataset have been taken using sensors dealing with the visible spectra but at two different times so that seasonal changes are preponderant. Compared to the two previous datasets, images are not rectified so that two images from the same pair have a different point of view. References [7,17] present interesting results on this dataset but give no details on feature extractors performance on single images: they only introduce an average accuracy of their respective method on the whole sequences matching. We apply the same evaluation protocol without sequential approach on this dataset and get the results presented in Table 3 and Figure 21.

Even if this dataset is quite challenging, PHROG feature permits an image retrieval task which is faulty approximately only one time in four and outperforms the other methods we have tested. PHROG presents the best AUC and the best precision when recall is low.

### 4.5. Experimentations on our Visible-SWIR Dataset

**Figure 22:**
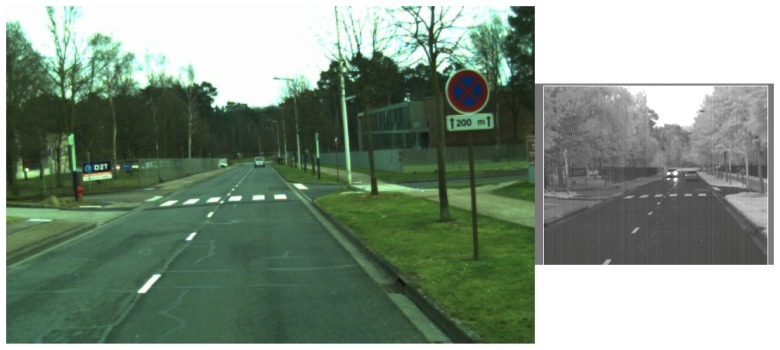
A Visible-SWIR pair from our dataset.

**Figure 23:**
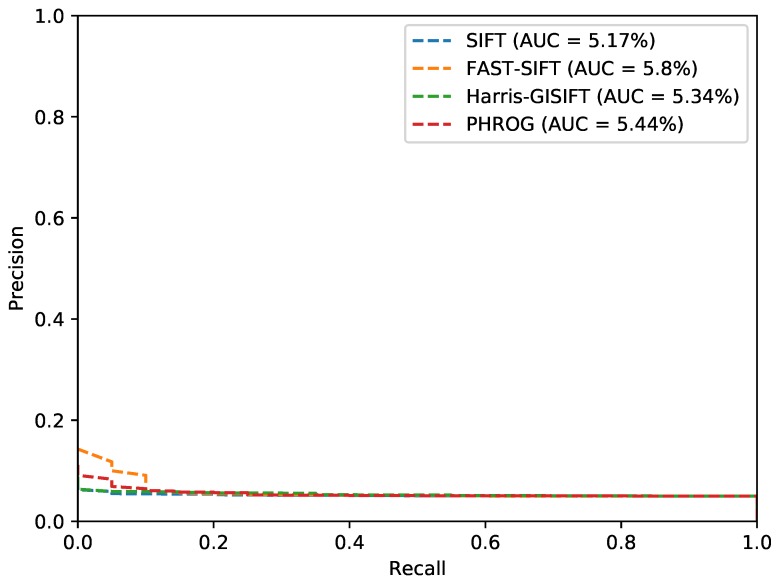
Precision-Recall curves from the “Long-Term” dataset and AUC (*Area Under the Curve*) for each method.

The last test case implies our own dataset. It has been made with a visible camera and a SWIR sensor. This dataset is much more challenging because image resolution and viewpoints are not the same. Evermore, one subset has been acquired several months later so that vegetation appearance is very different (with and without leaves). Figure 22 is an instance of this dataset. The SWIR set, with the smallest resolution, has been used as the memory first and the two modalities have been switched then. Results are presented in Table 4 and Figure 23. Our method shows again good results compared to the usual descriptors. A notable remark has to be made concerning the choice of the memory: results are better when the SWIR composes the memory than the visible one. We assume that the low and noisy resolution of the SWIR leads to less informative descriptors, and more general representations than with the visible set. Nevertheless, these results are poor in absolute terms, AUC are very low and no method raises significant results if we consider only the PR curves. We have taken this SWIR camera to make a step further in a highly constrained situation. Obviously, this camera is not the best choice to do when designing an embedded system, because of its low resolution compared to other devices, the noise generated on the images and its little interest in low light situations. Indeed, the visual place recognition problem requires additional process like filtering to refine matching with several incoming images for example.

## 5. Conclusions

Vision sensors have been investigated and valued for years in robotics, notably for outdoor applications and autonomous navigation. Studies and implementations have been led with several kind of cameras whose spectral sensibility differs. Each spectral range brings its strengths and weaknesses, resulting in technical choices made according to the final purpose: for instance, a color camera for road sign recognition, a near infrared one to enhance perception during foggy days and thermal infrared for pedestrian detection or even visual localization at night. Owing to such considerations, there is a diversity of systems processing variable kind of sensors data. In the pursuit of cooperating systems sharing their information collected in the surrounding environment, there is a need to design methods which are generalizable enough across spectral ranges.

Whereas a few works emphasize on these issues, we have considered in this paper the constraints introduced by the sensor invariance need, particularly when robotics systems embed cameras with different spectral ranges. We have studied image retrieval approaches between a visible dataset and an infrared one, being NIR, SWIR or LWIR. We have made these experimentations along with the typical constraints studied in the long-term visual place recognition literature: viewpoint and image resolution changes, long-term appearance evolution and illumination variations of the scene. The developed work in this paper proposes a new feature description method: based on a scoping study, we have determined that feature detectors dealing with contrasted shapes like Harris are more appropriated to a cross-modality repeatability. We have proposed a description pattern with a modified HOG feature which maximizes repeatability across different spectral ranges. This feature is then extracted at several scales in order to face the viewpoint changes.

We have evaluated our proposal on several well-known datasets used for multimodal feature benchmarking and one dedicated to visual place recognition. We have proposed another dataset with a SWIR sensor whose attributes (resolution for example) are more challenging. In that way, our paper presents experimental results on four kind of cameras with different spectral ranges, ensuring that our proposal do not over-fit a particular couple of visible/IR sensors but is rather generalized. We have noticed that some methods can be discriminant enough with a Bag-of-Word approach and are relevant for long-term localization. By the way, multimodal association still necessitates improvements, particularly when the sequences to be matched are more challenging due to the rise of additive constraints (perceptual changes, distant spectral ranges, inconsistent resolutions, noise, etc.). Our proposal shows interesting results which outperforms the traditional feature extractors. Local features still remain an interesting solution for SLAM-like issue: combined with data storage and retrieval methods (namely Bag-of-Words) for global localization and loop closure detection. As a future work, we plan to make this new image descriptor method part of a global SLAM system. This overall supervision will also permit to envision further an on-line visual localization process.

## Figures and Tables

**Table 1:** Results (matching ratio) on Visible-NIR dataset.

*Modality as Memory*	Dataset					
	Urban		Street		Country	
	Visible	NIR	Visible	NIR	Visible	NIR
SIFT-SIFT	96%	94%	78%	66%	40%	34%
SIFT-GISIFT	98%	94%	70%	64%	34%	32%
FAST-SIFT	**100%**	**100%**	**96%**	**96%**	**75%**	76%
Harris-SIFT	**100%**	98%	88%	92%	73%	61%
Harris-GISIFT	**100%**	**100%**	90%	90%	69%	67%
Our Method	**100%**	**100%**	**96%**	94%	73%	**80%**

**Table 2:** Results (matching ratio) on Barcelona dataset.

*Method*	Modality Used as Memory Set	
	LWIR	Visible
SIFT-SIFT	9%	9%
SIFT-GISIFT	11%	9%
FAST-SIFT	22%	9%
Harris-SIFT	18%	20%
Harris-GISIFT	52%	38%
Our method	**61%**	**56%**

**Table 3:** Results (matching ratio) on VPrice dataset.

*Method*	Efficiency
SIFT-SIFT	36%
SIFT-GISIFT	42%
FAST-SIFT	68%
Harris-SIFT	52%
Harris-GISIFT	47%
Our method	**73%**

**Table 4:** Results (matching ratio) on Long-Term dataset.

*Method*	Modality Used as Memory Set	
	SWIR	Visible
SIFT-SIFT	15%	5%
SIFT-GISIFT	20%	10%
FAST-SIFT	15%	**20%**
Harris-SIFT	15%	10%
Harris-GISIFT	25%	15%
Our method	**35%**	15%

## References

[1] Fox D., Ko J., Konolige K., Limketkai B., Schulz D., Stewart B. (2006). Distributed multirobot exploration and mapping. Proc. IEEE.

[2] Lowry S., Sünderhauf N., Newman P., Leonard J.J., Cox D., Corke P., Milford M.J. (2016). Visual place recognition: A survey. IEEE Trans. Robot..

[3] Cummins M., Newman P. (2011). Appearance-only SLAM at large scale with FAB-MAP 2.0. Int. J. Robot. Res..

[4] Brubaker M.A., Geiger A., Urtasun R. Lost! Leveraging the crowd for probabilistic visual self-localization. Proceedings of the IEEE Conference on Computer Vision and Pattern Recognition (CVPR).

[5] Chapoulie A., Rives P., Filliat D. A spherical representation for efficient visual loop closing. Proceedings of the IEEE International Conference on Computer Vision (ICCV) Workshops.

[6] Milford M.J., Wyeth G.F. SeqSLAM: Visual route-based navigation for sunny summer days and stormy winter nights. Proceedings of the IEEE International Conference on Robotics and Automation (ICRA).

[7] Neubert P., Sunderhauf N., Protzel P. Appearance change prediction for long-term navigation across seasons. Proceedings of the European Conference on Mobile Robots (ECMR).

[8] Naseer T., Spinello L., Burgard W., Stachniss C. Robust Visual Robot Localization Across Seasons using Network Flows. Proceedings of the 28th AAAI Conference on Artificial Intelligence.

[9] McManus C., Churchill W., Maddern W., Stewart A., Newman P. Shady Dealings: Robust, Long- Term Visual Localisation using Illumination Invariance. Proceedings of the IEEE International Conference on Robotics and Automation (ICRA).

[10] Mikolajczyk K., Schmid C. (2005). A performance evaluation of local descriptors. IEEE Trans. Pattern Anal. Mach. Intell..

[11] Heinly J., Dunn E., Frahm J.M. Comparative evaluation of binary features. Proceedings of the European Conference on Computer Vision (ECCV).

[12] Fraundorfer F., Scaramuzza D. (2012). Visual odometry: Part II: Matching, robustness, optimization, and applications. IEEE Robot. Autom. Mag..

[13] Hartley R., Zisserman A. (2003). Multiple View Geometry in Computer Vision.

[14] Fuentes-Pacheco J., Ruiz-Ascencio J., Rendón-Mancha J.M. (2015). Visual simultaneous localization and mapping: A survey. Artif. Intell. Rev..

[15] Garcia-Fidalgo E., Ortiz A. (2015). Vision-based topological mapping and localization methods: A survey. Robot. Autonom. Syst..

[16] Sivic J., Zisserman A. Video Google: A text retrieval approach to object matching in videos. Proceedings of the 9th IEEE International Conference on Computer Vision (ICCV).

[17] Mishkin D., Perdoch M., Matas J. Place recognition with WxBS retrieval. Proceedings of the IEEE Conference on Computer Vision and Pattern Recognition (CVPR), Workshop on Visual Place Recognition in Changing Environments.

[18] Koniusz P., Yan F., Mikolajczyk K. (2013). Comparison of mid-level feature coding approaches and pooling strategies in visual concept detection. Comput. Visi. Image Underst..

[19] Sünderhauf N., Shirazi S., Dayoub F., Upcroft B., Milford M. On the performance of convnet features for place recognition. Proceedings of the IEEE/RSJ International Conference on Intelligent Robots and Systems (IROS).

[20] Sunderhauf N., Shirazi S., Jacobson A., Dayoub F., Pepperell E., Upcroft B., Milford M. Place recognition with ConvNet landmarks: Viewpoint-robust, condition-robust, training-free. Proceedings of the Robotics: Science and Systems Conference (RSS).

[21] Weyand T., Kostrikov I., Philbin J. Planet-photo geolocation with convolutional neural networks. Proceedings of the European Conference on Computer Vision (ECCV).

[22] Aguilera C.A., Aguilera F.J., Sappa A.D., Aguilera C., Toledo R. Learning Cross-Spectral Similarity Measures with Deep Convolutional Neural Networks. Proceedings of the IEEE Conference on Computer Vision and Pattern Recognition (CVPR) Workshops.

[23] Davison A.J., Reid I.D., Molton N.D., Stasse O. (2007). MonoSLAM: Real-time single camera SLAM. IEEE Trans. Pattern Anal. Mach. Intell..

[24] Abrate F., Bona B., Indri M. Experimental EKF-based SLAM for Mini-rovers with IR Sensors Only. Proceedings of the 3rd European Conference on Mobile Robots (ECMR).

[25] Caron G., Dame A., Marchand E. (2014). Direct model based visual tracking and pose estimation using mutual information. Image Vis. Comput..

[26] Magnabosco M., Breckon T.P. (2013). Cross-spectral visual Simultaneous Localization And Mapping (SLAM) with sensor handover. Robot. Autonom. Syst..

[27] Maddern W., Vidas S. Towards robust night and day place recognition using visible and thermal imaging. Proceedings of the Robotics: Science and Systems Conference (RSS).

[28] Ricaurte P., Chilán C., Aguilera-Carrasco C.A., Vintimilla B.X., Sappa A.D. (2014). Feature Point Descriptors: Infrared and Visible Spectra. Sensors.

[29] Firmenichy D., Brown M., Susstrunk S. Multispectral interest points for RGB-NIR image registration. Proceedings of the 18th IEEE International Conference on Image Processing (ICIP).

[30] Mouats T., Aouf N. Multimodal stereo correspondence based on phase congruency and edge histogram descriptor. Proceedings of the 16th International Conference on Information Fusion (FUSION).

[31] Lowe D.G. (2004). Distinctive image features from scale-invariant keypoints. Int. J. Comput. Vis..

[32] Rosten E., Drummond T. Machine learning for high-speed corner detection. Proceedings of the European Conference on Computer Vision (ECCV).

[33] Mikolajczyk K., Tuytelaars T., Schmid C., Zisserman A., Matas J., Schaffalitzky F., Kadir T., Van Gool L. (2005). A comparison of affine region detectors. Int. J. Comput. Vis..

[34] Tuytelaars T., Mikolajczyk K. (2008). Local invariant feature detectors: A survey. Found. Trends® Comput. Graph. Vis..

[35] Harris C., Stephens M. A combined corner and edge detector. Proceedings of the Alvey Vision Conference.

[36] Shi J., Tomasi C. Good features to track. Proceedings of the IEEE Computer Society Conference on Computer Vision and Pattern Recognition (CVPR).

[37] Bay H., Ess A., Tuytelaars T., Van Gool L. (2008). Speeded-up robust features (SURF). Comput. Vis. Image Underst..

[38] Aguilera C.A., Sappa A.D., Toledo R. LGHD: A feature descriptor for matching across non-linear intensity variations. Proceedings of the IEEE International Conference on Image Processing (ICIP).

[39] Arandjelović R., Zisserman A. Three things everyone should know to improve object retrieval. Proceedings of the IEEE Conference on Computer Vision and Pattern Recognition (CVPR).

[40] Brown M., Susstrunk S. Multi-spectral SIFT for scene category recognition. Proceedings of the IEEE Conference on Computer Vision and Pattern Recognition (CVPR).

